# Abdominal ectopic pregnancy after in vitro fertilization and single embryo transfer: a case report and systematic review

**DOI:** 10.1186/s12958-016-0201-x

**Published:** 2016-10-19

**Authors:** Nicole Yoder, Reshef Tal, J. Ryan Martin

**Affiliations:** Division of Reproductive Endocrinology & Infertility, Department of Obstetrics, Gynecology, & Reproductive Sciences, Yale University School of Medicine, 333 Cedar Street, New Haven, CT 06510 USA

**Keywords:** Abdominal pregnancy, Ectopic pregnancy, In vitro fertilization, IVF-ET

## Abstract

**Background:**

Ectopic pregnancy is the leading cause of maternal morbidity and mortality during the first trimester and the incidence increases dramatically with assisted-reproductive technology (ART), occurring in approximately 1.5–2.1 % of patients undergoing in-vitro fertilization (IVF). Abdominal ectopic pregnancy is a rare yet clinically significant form of ectopic pregnancy due to potentially high maternal morbidity. While risk factors for ectopic pregnancy after IVF have been studied, very little is known about risk factors specific for abdominal ectopic pregnancy. We present a case of a 30 year-old woman who had an abdominal ectopic pregnancy following IVF and elective single embryo transfer, which was diagnosed and managed by laparoscopy. We performed a systematic literature search to identify case reports of abdominal or heterotopic abdominal ectopic pregnancies after IVF. A total of 28 cases were identified.

**Results:**

Patients’ ages ranged from 23 to 38 (Mean 33.2, S.D. = 3.2). Infertility causes included tubal factor (46 %), endometriosis (14 %), male factor (14 %), pelvic adhesive disease (7 %), structural/DES exposure (7 %), and unexplained infertility (14 %). A history of ectopic pregnancy was identified in 39 % of cases. A history of tubal surgery was identified in 50 % of cases, 32 % cases having had bilateral salpingectomy. Transfer of two embryos or more (79 %) and fresh embryo transfer (71 %) were reported in the majority of cases. Heterotopic abdominal pregnancy occurred in 46 % of cases while 54 % were abdominal ectopic pregnancies.

**Conclusions:**

Our systematic review has revealed several trends in reported cases of abdominal ectopic pregnancy after IVF including tubal factor infertility, history of tubal ectopic and tubal surgery, higher number of embryos transferred, and fresh embryo transfers. These are consistent with known risk factors for ectopic pregnancy following IVF. Further research focusing on more homogenous population may help in better characterizing this rare IVF complication and its risks.

## Background

Ectopic pregnancy is the leading cause of maternal morbidity and mortality during the first trimester and the incidence increases dramatically with assisted reproductive technology (ART), occurring in approximately 1.5–2.1% of patients undergoing IVF [1, 2]. The majority of ectopic pregnancies from either IVF or spontaneous pregnancy occur within the fallopian tubes, but implantation may occur in other locations such as the cervix, ovary, or abdomen [3]. Abdominal ectopic pregnancies are a very rare form of ectopic pregnancy, yet are clinically significant due to their potential for high morbidity and often atypical presentation [4].

Recent studies have attempted to identify risk factors for ectopic pregnancy after IVF. Suggested risk factors include infertility due to tubal factor, endometriosis, transfer at blastocyst stage, higher number of embryos transferred, decreased endometrial thickness, variation in culture media, and fresh embryo transfer [5–9]. However, very little data exists regarding risk factors for abdominal ectopic pregnancy after IVF.

In this case study, we report an abdominal ectopic pregnancy after IVF with fresh single embryo transfer. We also performed a systematic review of the literature for known cases of abdominal ectopic pregnancy after IVF and provide detailed characterization of these patients and risk factors for this rare complication.

### Case description

The patient was a 30-year-old G2P0010 who presented to our fertility center seeking fertility treatment. She had a medical history of polycystic ovarian syndrome (PCOS) and her partner had a diagnosis of male factor infertility. She had no prior surgical history, no known allergies, and medications included prenatal vitamins. She denied any history of sexually transmitted infections and had a normal hysterosalpingogram and saline sonohysterogram. Her first IVF cycle with an elective single embryo transfer resulted in a negative pregnancy test. Her second IVF cycle used a GnRH antagonist stimulation protocol and she was triggered with Ovidrel on stimulation day 12. Twenty-two oocytes were retrieved. On day five a single fresh blastocyst was transferred using a pass through technique under ultrasound guidance. A stiff outer sheath was introduced through the cervix and past the internal os. A soft tipped catheter containing the embryo was advanced through the outer sheath and the embryo was expelled into the uterine cavity approximately 1.5 cm from the uterine fundus with good visualization. Beta hCG was positive on post-transfer day 9 and serial beta hCG values were monitored and continued to rise appropriately (Table 1). On day 28 after embryo transfer, the patient underwent a transvaginal ultrasound (TVUS) in the office that did not identify an intrauterine pregnancy (IUP) or any abnormal adnexal structures. She was asymptomatic with no vaginal bleeding or abdominal pain. The patient was sent for a more comprehensive ultrasound evaluation at the associated Maternal Fetal Medicine unit and another beta hCG value was obtained. Repeat scan similarly failed to identify an IUP or visualize an ectopic pregnancy. The beta hCG was 12,400 pg/mL. Given the high beta hCG value in the absence of an IUP, the patient was counseled and advised to take methotrexate treatment for presumed ectopic pregnancy of unknown location. One day later (day 29), she received an intramuscular dose of 83 mg (50 mg/m^2^ body surface area) methotrexate with plans to follow up with repeat beta hCG and TVUS.

**Table 1:** Beta hCG level and timeline of events

Day	Beta HCG pg/mL	Event
−5		Oocyte retrieval, ICSI
0		Day 5 single embryo transfer
9	28.7	
11	45.5	
13	130	
15	382	
17	991	
19	2020	
28	12,400	Sac Check - No IUP or adnexal abnormalities
29	13,000	Methotrexate given
32	20,000	
33		TVUS - Right adnexal mass with gestational sac and fetal cardiac activity
34		Diagnostic laparoscopy - Abdominal ectopic

**Fig. 1:**
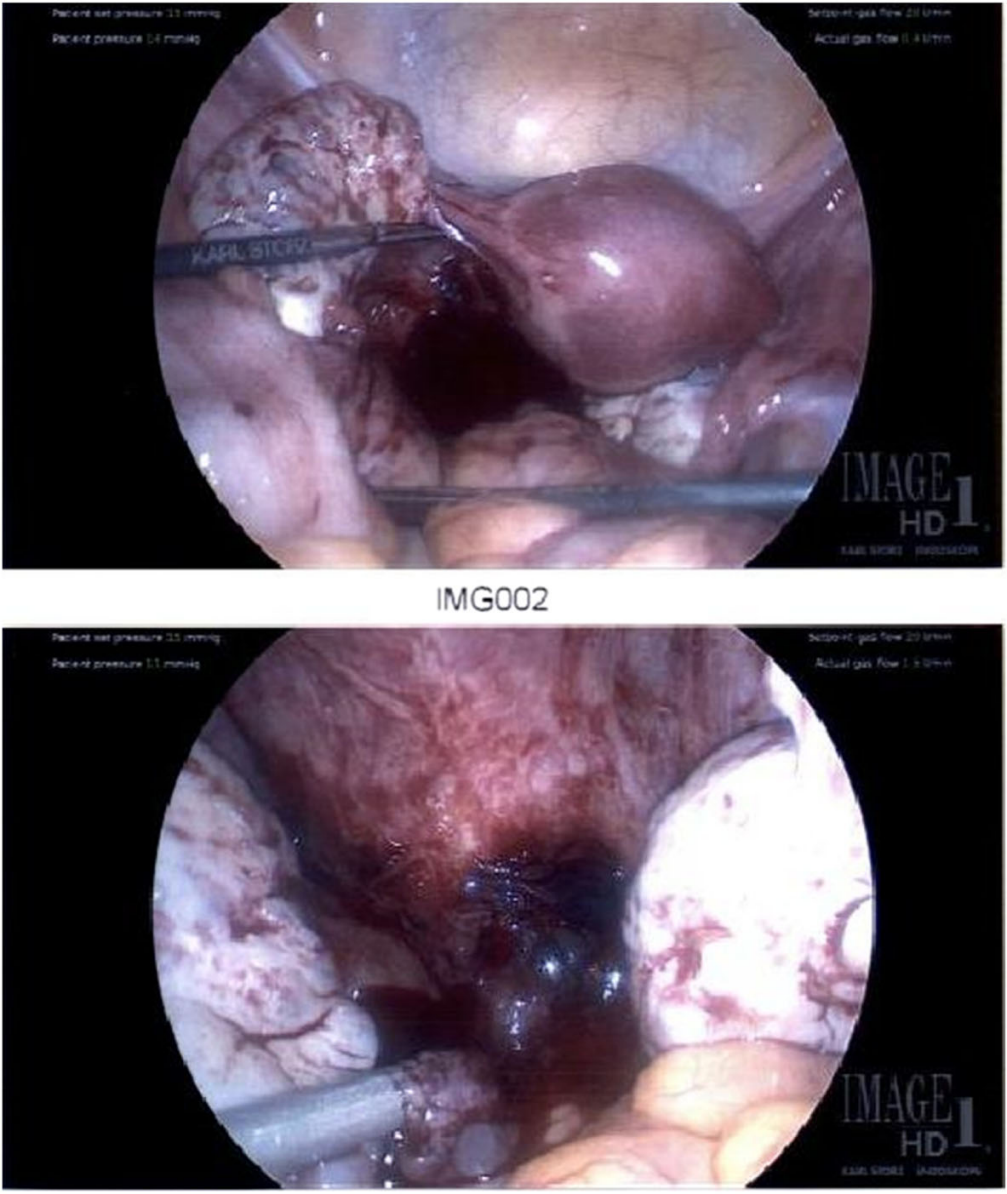
Diagnostic laparoscopy demonstrating hemoperitoneum (*top image*) and products of conception implanted in the posterior cul-de-sac (*bottom image*)

Four days after methotrexate administration, repeat beta hCG level continued to rise (20,000 pg/mL) and an ultrasound performed 1 day later demonstrated a right adnexal mass with a yolk sac, fetal pole, and fetal cardiac activity. The decision was made to proceed with diagnostic laparoscopy for treatment of ectopic pregnancy after failure of methotrexate therapy. The patient continued to be asymptomatic with no vaginal bleeding or abdominal pain. Diagnostic laparoscopy was performed on day 34 post-embryo transfer. The operative findings were significant for minimal hemoperitoneum (<50 mL) and products of conception were noted to be implanted on the peritoneum of the posterior cul-de-sac medial to the left uterosacral ligament (Fig. 1). The products of conception were removed using graspers without difficulty and hemostasis was obtained with electrocautery and surgicel. All other pelvic organs including uterus and bilateral ovaries and tubes appeared grossly normal in appearance.

### Systematic review of the literature

A systematic literature review was performed with the aim of identifying all other case reports of abdominal ectopic pregnancies after IVF. The literature search was performed using PubMed, Google Scholar, and EMBASE without language restriction encompassing publications until July 2016. Search terms used included ‘IVF’, ‘ectopic pregnancy’, ‘abdominal ectopic pregnancy’, and ‘heterotopic pregnancy’. To the best of our knowledge, all reported cases and available data are summarized in Table 2.

**Table 2:** Abdominal ectopic case reports

Author (year)	Age/Parity	Infertility etiology	Other pertinent history	Priorectopic	Stimulation Protocol	Egg #	ET no./timing	Fresh/Frozen ET	Max HCG level (mIU/ml)	Location (E/H)	Stage at diagnosis	Rupture?	Intervention	Outcome
Oehniger (1988) [23]	35 yo G0P0	Endometriosis	Laparotomy x 2, left salpingectomy, frozen pelvis; Right hydrosalpinx with partial obstruction	No	FSH/Pergonal (hMG/hCG), hCG trigger	4	4 42–44 h	Fresh	NA	Sigmoid mesentery (E)	~41 days PT	No	Exploratory Laparotomy	Removal of pregnancy tissue by laparotomy
Bassil (1991) [24]	33 yo NA	Male factor	NA	NA	Clomid/hMG, hCG trigger	6	4 NA	Fresh	NA	Posterior uterus, broad ligament (H)	19 weeks gestation	No	Laparotomy, right adnexectomy	Delivery of viable twins at 34 weeks
Ferland (1991) [25]	32 yo G4P0030	DES exposure, secondary infertility	Right salpingectomy, left hydrosalpinx	Tubal ectopic	Long protocol w/GnRH agonist	7	3 Day 2 ET	Fresh	19,450	Retroperitoneal (E)	37 days PT	Yes	Laparotomy, left salpingectomy	
Ragni (1991) [26]	32 yo G1P0010	Pelvic adhesive disease	Right adnexectomy, hysteropexy	Tubal ectopic	Long protocol w/GnRH agonist	4	3 Day 2 ET	Fresh	NA	Right adnexa (H)	12 weeks gestation	No	Selective reduction of abdominal pregnancy, laparotomy	Laparotomy for resorbing abdominal pregnancy, SAB of IUP at 16 weeks
Balmaceda (1993) [27]	33 yo G3P1021	Tubal	Right salpingectomy, left salpingostomy	Tubal Ectopic *x*2	Short protocol, w/GnRH agonist	15	4 Day 4 ET	Fresh	4651	Abdominal - broad ligament (E)	30 days PT	No	Laparoscopy, salpingectomy	Laparoscopic removal of abdominal ectopic, left salpingectomy
Fisch (1995) [28]	32 yo G2P0020	Tubal	Bilateral salpingectomy	Tubal ectopic *x*2	Long protocol w/GnRH agonist	5	3 NA	Fresh	NA	Ileum, left uterine cornua (H)	10 weeks gestation	Yes	Gastrostoscopy, sigmoidoscopy, Tc scan, angiography, D&C, tagged RBC scan, Laparotomy	Laparotomy for abdominal ectopic, D&C for incomplete AB of IUP
DelRosario (1996) [29]	33 yo G1P1001	Tubal	Breast Cancer	No	NA	NA	4 NA	Frozen	563	Bladder (E)	75 days PT	Yes	Methotrexate, laparoscopy	Laparoscopic removal of pregnancy tissue
Fisch (1996) [11]	38 yo G2P0020	Tubal	Laparoscopic Salpingectomy *x*2, 8th IVF cycle	Tubal ectopic x 2	Long protocol w/GnRH agonist	14	4 Day 3 ET	Fresh	1730	Broad Ligament (E)	21 days PT	Yes	Exploratory Laparotomy	Removal of pregnancy tissue by laparotomy
Moonen-Delarue (1996) [30]	23 yo G2P0020	Pelvic adhesive disease	Right salpingectomy	Tubal and abdominal ectopic	NA	NA	NA NA	Fresh	NA	Abdominal - uterine fundus (E)	28 weeks	Placental abruption	Laparotomy	Fetal demise of abdominal ectopic @ 28 weeks
Pisarska (1998) [31]	35 yo G2P0020	Unexplained	NA	No	Long protocol w/GnRH agonist	9	6 NA	Fresh	6004	Bladder serosa (H)	6 weeks gestation	No	Diagnostic laparoscopy	Laparoscopic removal of ectopic pregnancy (bladder), term delivery of IUP
Deshpande (1999) [32]	33 yo G1P0010	Endometriosis	Endometriosis, left salpingectomy, Patent right tube	No	Long protocol w/GNRH agonist	8	2 Day 3 ET	Fresh	55,560	Twin pregnancy in broad ligament (H)	7 weeks PT	No	Laparotomy	Removal of twin ectopic pregnancy by laparotomy at 7 weeks
Scheiber (1999) [33]	37 yo G3P0030	Tubal factor EndometriosisDOR	Salpingostomy, donor oocytes	Tubal ectopic	NA	NA	2 Day 3 ET	Frozen	NA	Abdominal (H)	8.5 weeks PT	No	KCl selective reduction of abdominal pregnancy	Selective reduction of abdominal pregnancy, full term viable IUP
Dmowski (2002) [34]	34 yo G0P0	Tubal	Bilateral Salpingectomy	No	Long protocol w/GnRH agonist	15	3 Day 3 ET	Fresh	38,635	Retroperitoneal pancreatic (E)	41 days PT	Yes	Laparotomy	Retroperitoneal subpancreatic ectopic removed by laparotomy
Jain (2002) [35]	29 yo G0P0	Unexplained	NA	No	NA	NA	2 NA	NA	NA	Pouch of Douglas (H)	9 weeks PT	NA	Laparotomy at 4w weeks (no IUP seen), selective reduction of ectopic at 13 weeks	Selective reduction of abdominal ectopic, removal by laparotomy, SAB of IUP
Cormio (2003) [36]	30 yo G2P0020	Tubal	Bilateral salpingectomy	Tubal ectopic *x*2	Menotropins, hCG trigger	7	4 Day 3 ET	Fresh	256,400	Omentum, uterine fundus (H)	13 weeks gestation	No	Laparotomy	Laparotomy for abdominal ectopic; Live IUP delivered at 36 weeks
Reid (2003) [37]	28 yo G5P1041	Tubal	bilateral salpingectomy	Tubal ectopic x3	NA	NA	3 NA	NA	5500	Retroperitoneal, iliac bifurcation (E)	63 days PT	NA	Laparotomy	Removal of ectopic via laparotomy
Kitade (2005) [38]	37 yo G0P0	Unexplained	NA	No	Long protocol w/GnRH agonist	12	3 Day 3 ET	Fresh	45,896	Splenic and Tubal (H)	34 days PT (tubal), 46 day PT (splenic)	Tubal - No, Splenic - Yes	1) Laparoscopic salpingectomy 2) Exploratory laparotomy	Removal of tubal ectopic by laparoscopy, removal of splenic ectopic by laparotomy (12 days later)
Ali (2006) [39]	35 NA	Tubal	Pelvic adhesions	No	NA	11	1 NA	Fresh	1524	Tube with Omental/peritoneal trophoblastic tissue (H)	3 weeks PT - tubal ectopic; 5 weeks PT – omental tissue	No	Laparoscopic salpingectomy; Laparocopic removal of omental/peritoneal trophoblastic tissue	Removal of tubal and peritoneal/omental pregnancy tissue by 2 laparoscopies
Apantaku (2006) [40]	33 G3P1021	Tubal	Bilateral salpingectomy	Tubal ectopic *x*2	NA	NA	2 NA	Fresh	NA	Right adnexa (E)	6 weeks PT	No	Laparoscopy	Laparoscopic removal of pregnancy tissue
Knopman (2007) [41]	37 yo G4P0040	Unexplained	NA	No	GnRH antagonist	9	2 Day 5 ET	Fresh	1023	Posterior cul-de-sac (H)	7 weeks, nonviable IUP; 9 weeks ectopic	Yes	Laparoscopy	D&C for non-viable IUP; Laparoscopy for abdominal ectopic
Shih (2007) [42]	33 yo G0P0	Male Factor	Patent tubes	No	Long protocol w/GnRH agonist	4	NA NA	Fresh	901	Cul-de-sac (E)	28 days PT	No	Laparoscopy converted to laparotomy	Removal of pregnancy tissue by laparotomy
Shojai (2007) [43]	35 yo G0P0	Structural, DES exposure	NA	No	NA	NA	3 NA	NA	NA	Abdominal - uterine fundus (H)	21 weeks gestation	No	Laparotomy	Delivery of viable twins at 32 weeks
Iwama (2008) [44]	31 yo G1P0010	Tubal	Right Salpingectomy for tubal ectopic after IVF, left salpingectomy for hydrosalpinx	Tubal ectopic	NA	NA	3 Day 3 ET	Fresh	45, 369	Inferior Vena Cava/Retroperitoneal (E)	32 days PT: PUL; 53 days PT: retroperitoneal ectopic	Yes	D&C, MTX, Diagnostic laparoscopy, repeat MTX, Exploratory laparotomy	Ruptured retroperitoneal ectopic, removed by laparotomy
Hyvarinen (2009) [45]	NA NA	NA	NA	NA	NA	NA	NA NA	NA	NA	Abdominal (E)	30 weeks gestation	No	Laparotomy	Delivery of viable fetus at 30 weeks
Zacche (2011) [46]	36 G1P1	Tubal	Bilateral Salpingectomy, PID	No	NA	NA	2 NA	Fresh	NA	Abdominal (H)	32 weeks at Cesarean Delivery	No	Laparotomy, hysterectomy	Viable twin pregnancies at 32 weeks; Hysterectomy
Angelova (2015) [47]	33 NA	Male Factor	Obturated left tube	NA	Short protocol, w/GnRH antagonist	NA	2 Day 3 ET	Fresh	NA	Abdominal - vesicouterine junction (E)	23 days PT	No	Laparoscopy	Laparoscopic removal of pregnancy tissue
Dalmia (2015) [48]	37 G1P0010	EndometriosisTubal factor	Bilateral salpingectomy for hydrosalpinx	NA	NA	NA	NA NA	NA	21,730	Left adnexa (E)	2 weeks PT	No	Mini-laparotomy	Removal of ectopic via laparotomy
Koyama (2015) [49]	32 G5P1	Male Factor	NA	No	NA	NA	1 NA	Frozen	14,800	Retroperitoneal (E)	10 weeks gestation	NA	Laparoscopy	Laparoscopic removal of pregnancy tissue

*Abbreviations*: *AB* Abortion, *D&C* Dilation and curettage, *DES* Diethylstilbestrol, *E* Ectopic, *FSH* Follicle stimulating hormone, *GnRH* Gonadotropin-releasing hormone, *H* Heterotopic, *hCG* Human chorionic gonadotropin, *hMG* Human menopausal gonadotropin, *HSG* Hysterosalpingogram, *IUP* Intrauterine pregnancy, *IVF* In vitro fertilization, *KCl* Potassium chloride, *MTX* Methotrexate, *NA* Not available, *PID* Pelvic inflammatory disease, *PT* Post transfer, *RBC* Red blood cell, *Tc* Technetium, *SAB* Spontaneous abortion

## Results

A total of 28 cases of abdominal ectopic pregnancy after IVF were identified. The age of patients ranged from 23 to 38 yo (Mean = 33.2 S.D. = 3.2), with no age reported in 1 case. Infertility causes included tubal factor in 13 (46 %) cases, endometriosis in 4 (14 %) cases, male factor in 4 (14 %) cases, pelvic adhesive disease in 2 (7 %) cases, structural/DES exposure in 2 (7 %) cases, unexplained in 4 (14 %) cases, and one case did not specify the cause. Overall, anatomic/structural factors accounted for 17 (61 %) of the cases. A history of ectopic pregnancy was identified in 11 (39 %) cases. History of tubal surgery had been described in 14 (50 %) cases, 9 (32 %) of which were bilateral salpingectomy. Transfer of more than two embryos was reported in 15 (54 %) cases, two embryos were transferred in 7 (25 %) cases, while single embryo transfer was reported in only two (7 %) cases. No information about number of embryos transferred was available in 4 (14 %) cases. Fresh embryo transfer accounted for 20 (71 %) cases, frozen embryo transfer in 3 (11 %) cases, and 5 (18 %) cases did not specify fresh versus frozen embryo transfer. Heterotopic abdominal pregnancy occurred in 13 (46 %) cases, and 15 (54 %) were abdominal ectopic pregnancies. Notable cases include 5 retroperitoneal ectopic pregnancies, an abdominal fetal demise at 28 weeks, and 4 cases of viable abdominal pregnancies at 30 weeks, 32 weeks (two cases), and 34 weeks gestation.

## Discussion

Abdominal ectopic pregnancies comprise less than 1 % of all ectopic pregnancies, yet have a maternal mortality rate eight times greater than tubal ectopic pregnancies [10]. For this reason, early recognition and treatment is crucial in the setting of abdominal ectopic pregnancy. The case presented demonstrates the diagnostic challenge of abdominal ectopic, as the patient’s beta hCG values followed a normal rise and the patient remained asymptomatic up to the point of diagnostic laparoscopy. Transvaginal ultrasound did not visualize the ectopic pregnancy until the beta hCG value was 20,000 pg/mL, which is far beyond the usual discriminatory zone. This atypical presentation of an ectopic pregnancy highlights the need to consider abdominal ectopic pregnancy in the differential of any pregnancy of unknown location after IVF, especially in the setting of non-diagnostic transvaginal ultrasound.

There appears to be an increased rate of ectopic pregnancies after ART when compared to rates in spontaneous pregnancy [11]. As the number of IVF procedures performed continues to rise, the incidence of ectopic and abdominal ectopic pregnancy will likely also rise. While there are still relatively few reported cases of abdominal ectopic pregnancies after IVF, our systematic review demonstrates several trends among reported cases. First, the majority of cases (61 %) report a history of anatomic/structural infertility etiology with history of tubal factor infertility (TFI) (46 %) being the most common. This is consistent with TFI being a known risk factor for ectopic pregnancy following IVF. One study that examined the risk factors for EP following IVF in 712 women reported an odds ratio (OR) of 3.99 (95 % CI: 1.23 to 12.98) for women with TFI compared to those with other infertility causes [12]. In a larger, more recent study of 553,577 ART cycles in the US, among all infertility diagnoses, TFI was the only one significantly associated with increased risk for ectopic pregnancy (adjusted relative risk (RR) 1.25, 95 % CI 1.16–1.35) [13]. In addition, history of tubal ectopic pregnancy was particularly common, being reported in 37 % of the abdominal ectopic cases. This also appears to be consistent with the general ART-associated EP literature. A retrospective study that measured the risk of EP following IVF in 181 women with a previous ectopic demonstrated a 45-fold higher risk of recurrence when compared with 377 women with other causes of infertility. The authors reported that the prevalence of EP was 8.95 % compared with 0.75 % in the control group [14]. History of prior tubal surgery was also particularly common (50 %) among abdominal ectopic cases in our systematic review. A history of tubal/pelvic surgery is another major risk factor for the development of EP following IVF. Odds ratio for developing EP was 8.52 (95 % CI: 5.91–12.27) for prior adnexal surgery, 11.02 (95 % CI: 5.49–22.15) for a previous tubal infertility surgery, 5.16 (95 % CI: 1.25–21.21) for prior surgery for endometriosis and 17.70 (95 % CI: 8.11–38.66) for a previous abdominal/pelvic surgery [12, 15, 16]. Interestingly, bilateral salpingectomy was the most common tubal surgery reported in our case review. While the exact mechanism of abdominal ectopic after bilateral salpingectomy remains unclear, many authors have proposed that it may be due to the development of a micro-fistulous tract after salpingectomy. Uterine perforation during embryo transfer has also been suggested as a mechanism for abdominal ectopic pregnancy, and embryo transfer technique has been related to overall EP risk after IVF. Aspects of the transfer that may increase risk of EP include large volume of transfer media, induction of abnormal uterine contractions, and location of embryo transfer in relation to the uterine fundus [9]. These factors have all been associated with retrograde flow of both transfer media and the embryo toward the fallopian tubes. Many suggestions have been made regarding optimal transfer location within the endometrium, ranging from 5 to 20 mm from the fundal surface, while others recommend “mid-cavity” location to avoid proximity to the fallopian tubes [17–19].

Other trends identified in our systematic review include >1 embryo transferred (reported in 79 % of cases) and a large number of heterotopic abdominal pregnancy (reported in 46 % of cases). Multiple embryo transfer has always been associated with increased risk of EP with transfer of two or less embryos carrying lower risk than after three or more embryos [20]. In the setting of multiple embryo transfers, identification of an intrauterine pregnancy often leads to delayed diagnosis of abdominal pregnancy in the absence of clinical symptoms. Among the heterotopic cases, 4 reported a 2 week delay in diagnosis of the abdominal ectopic from the time of suspected ectopic, and 5 cases did not identify the abdominal ectopic until beyond the 12th week of pregnancy. Unfortunately, this type of delayed diagnosis has the potential to lead to significantly morbid outcomes. In our review, four cases of viable abdominal pregnancies were identified, which is an extremely rare outcome. Three of these cases were identified at 19 weeks or beyond, and all three had attachment of the abdominal placenta to the peritoneal surface of the uterus without involvement of other abdominal organs. Placental attachment to the uterus has previously been associated with viability of abdominal pregnancies [21], and with a relatively lower risk of bleeding and lower likelihood of fetal growth retardation [22].

Finally, abdominal ectopic pregnancies were far more common in fresh embryo transfer (71 % of cases) than frozen embryo transfer (11 % of cases). This may be due to the fact that frozen embryo transfer has become widely used only recently, and we may begin to see higher frequency with frozen embryo transfers over time. However, several recent studies indicate that ectopic pregnancy rates are higher for fresh as compared to frozen IVF cycles [1, 6].

A limitation of this review is the heterogeneity of reported cases and IVF practices which encompass several decades. Further research focusing on more homogenous population may help in better characterizing this rare IVF complication.

## Conclusions

In conclusion, ectopic pregnancy, including abdominal ectopic, is a known risk of IVF. The case reported highlights the diagnostic challenges behind this rare form of ectopic pregnancy, and the need to keep it in the differential in atypical ectopic presentations. Our systematic literature review has revealed several trends in reported cases of abdominal ectopic pregnancy after IVF including tubal factor infertility, history of tubal ectopic and tubal surgery, higher number of embryos transferred, and fresh embryo transfers. These are consistent with known risk factors for ectopic pregnancy following IVF.

## Abbreviations

AB: Abortion
ART: Assisted reproduction technologies
D&C: Dilation and curettage
DES: Diethylstilbestrol
E: Ectopic
FSH: Follicle stimulating hormone
GnRH: Gonadotropin-releasing hormone
H: Heterotopic
hCG: Human chorionic gonadotropin
hMG: Human menopausal gonadotropin
HSG: Hysterosalpingogram
IUP: Intrauterine pregnancy
IVF: In vitro fertilization
KCl: Potassium chloride
MTX: Methotrexate
NA: Not available
PID: Pelvic inflammatory disease
PT: Post transfer
RBC: Red blood cell
SAB: Spontaneous abortion
Tc: Technetium
